# Inhibition of TGF-*β*1 Signaling by IL-15: A Novel Role for IL-15 in the Control of Renal Epithelial-Mesenchymal Transition: IL-15 Counteracts TGF-*β*1-Induced EMT in Renal Fibrosis

**DOI:** 10.1155/2019/9151394

**Published:** 2019-07-07

**Authors:** Aurore Devocelle, Lola Lecru, Hélène François, Christophe Desterke, Cindy Gallerne, Pierre Eid, Oberlin Estelle, Bruno Azzarone, Julien Giron-Michel

**Affiliations:** ^1^INSERM UMRS 1197, Hôpital Paul Brousse, 94807 Villejuif Cedex, France; ^2^Paris-Saclay University, France; ^3^IFRNT, AP-HP Bicêtre Hospital, 94270 le Kremlin-Bicêtre, France; ^4^UMS33, Paris-Saclay University, Paul Brousse Hospital, Villejuif, France; ^5^Immunology Research Area, IRCCS Bambino Gesù Pediatric Hospital, Rome, Italy

## Abstract

Renal tubulointerstitial fibrosis is the final common pathway in end-stage renal disease and is characterized by aberrant accumulation of extracellular matrix (ECM) components secreted by myofibroblasts. Tubular type 2 EMT, induced by TGF-*β*, plays an important role in renal fibrosis, by participating directly or indirectly in myofibroblasts generation. TGF-*β*1-induced apoptosis and fibrosis in experimental chronic murine kidney diseases are concomitantly associated with an intrarenal decreased expression of the IL-15 survival factor. Since IL-15 counteracts TGF-*β*1 effects in different cell models, we analyzed whether (1) human chronic inflammatory nephropathies evolving towards fibrosis could be also characterized by a weak intrarenal IL-15 expression and (2) IL-15 could inhibit epithelial-mesenchymal transition (EMT) and excess matrix deposition in human renal proximal tubular epithelial cells (RPTEC). Our data show that different human chronic kidney diseases are characterized by a strong decreased expression of intrarenal IL-15, which is particularly relevant in diabetic nephropathy, in which type 2 tubular EMT plays an important role in fibrosis. Moreover, primary epithelial tubular cultures deprived of growth supplements rapidly produce active TGF-*β*1 inducing a “spontaneous” EMT process characterized by the loss of membrane-bound IL-15 (mbIL-15) expression. Both “spontaneous” EMT and recombinant human (rh) TGF-*β*1-induced EMT models can be inhibited by treating RPTEC and HK2 cells with rhIL-15. Through a long-lasting phospho-c-jun activation, IL-15 inhibits rhTGF-*β*1-induced Snail1 expression, the master inducer of EMT, and blocks TGF-*β*1-induced tubular EMT and downstream collagen synthesis. In conclusion, our data suggest that intrarenal IL-15 could be a natural inhibitor of TGF-*β* in human kidney able to guarantee epithelial homeostasis and to prevent EMT process. Thus, both* in vivo* and* in vitro* an unbalance in intrarenal IL-15 and TGF-*β*1 levels could render RPTEC cells more prone to undergo EMT process. Exogenous IL-15 treatment could be beneficial in some human nephropathies such as diabetic nephropathy.

## 1. Introduction

Epithelial-to-mesenchymal transition (EMT) is a critical process that occurs both in normal development and in pathological settings, where epithelial cells lose their epithelial nature and gain mesenchymal characteristics [1]. EMT has been classified into three types. Type 1 EMT is associated with embryogenesis and occurs during normal organogenesis as an intermediate step leading, through the mesenchymal to epithelial transition (MET) process, to the generation of more mature epithelia. Type 2 EMT is associated with tissue repair responses generating myofibroblasts from epithelia to repair injured tissues in parenchymal organs; however, the reparatory process may degenerate into fibrosis [2]. Type 3 EMT is related to malignancy, where neoplastic cells acquire a migratory phenotype invading surrounding tissues and favouring the metastatic process [2].

Renal interstitial fibrosis is the final result of chronic inflammatory processes during which the interplay among different cellular components and a complex network of signaling pathways leads to the development of renal myofibroblasts responsible for an excessive accumulation of extracellular matrix (ECM) components, a major and common hallmark of different chronic kidney disease (CKD) [3]. However, the origin of myofibroblasts, which could derive from renal epithelial/endothelial cells, interstitial fibroblastic cells, or mesenchymal pericytes, remains the subject of controversial debates [4–8]. Results have conflicted, assigning either an important or a negligible role to tubular type 2 EMT in myofibroblasts generation [9–12], whose intervention is however limited to diabetic nephropathy (DN) [13–15]. In this context, a recent study reconciles conflicting data on role of EMT, showing that a partial and reversible tubular EMT, induced by Snail1 reactivation, relays signals to the interstitium to promote myofibroblast differentiation from cells of renal or extrarenal origin, demonstrating a new role of EMT in renal fibrosis [16]. Moreover, TGF-*β*1–induced expression of Twist1 or Snail1 induces a partial EMT that leads to G2 arrest of tubular epithelial cells, limiting their potential for repair and regeneration [11].

Type 2 EMT is triggered by a variety of soluble factors. The most profibrogenic one is intrarenal TGF-*β*1, whose production by renal cells has been linked to the development different nephropathies, membranous nephropathies, and other chronic renal diseases [17]. TGF-*β*1 exerts its effect through either Smad or non-Smad pathways and induces in cultured renal epithelial cells the acquisition of a spindle shape morphology associated with the expression of myofibroblastic markers and loss of epithelial ones [18].

On the other hand, numerous endogenous antifibrotic factors involved in kidney repair and regeneration have been identified. These regulators particularly morphogenic protein 7 (BMP-7) and hepatocyte growth factor (HGF) inhibit in particular tubular EMT interfering with TGF-*β*1-signaling. However, expression of both factors has been reported to be downregulated in chronic kidney injury [4, 19]. An important strategy for antifibrotic therapy would be to increase or restore the expression of antifibrotic factors in the diseased kidney.

Moreover, according to recent studies, it appears that more factors play a role in the regulation of EMT, illustrating the difficulty in understanding this complex process [14]. In this context, it is therefore possible that regulatory function of additional intrarenal factors has been underestimated, such as interleukin-15 (IL-15) which acts, through autocrine loops, as a powerful survival [20, 21] and homeostatic factor [22, 23] for renal epithelial cells. Indeed, experiments in IL-15-/- and IL-15R*α*-/- mice show that intrarenal IL-15, present both as secreted and membrane-bound, forms (mbIL-15) anchored through the IL-15R*α* chain and protects kidney epithelial cells, counteracting apoptosis, and inflammation during nephritis [20, 21]. In addition, in several murine experimental nephropathies there is a sharp and rapid decrease of intrarenal IL-15, which is detrimental to renal cell survival and kidney function during pathological stress [20, 21]. On the other hand, our recent data showed that IL-15 induces the differentiation of CD105+ renal cancer stem cells into epithelial cells that share several properties with normal tubular cells owing to the acquired production of their own IL-15 [24]. Based on these data, intrarenal IL-15 appears to be a powerful endogenous factor of epithelial homeostasis. In addition, IL-15 is able in addition to counteract TGF-*β*1 signal and effects in different cell models [25–29].

In this manuscript, we have explored the intrarenal IL-15 expression in different human inflammatory nephropathies evolving towards renal fibrosis and observed an important decreased expression, as shown in murine models [20, 21]. In addition, we also showed the capacity of recombinant human IL-15 (rhIL-15) to counteract TGF-*β*1-induced type 2 EMT in two classical human cellular models: cultures of primary proximal tubular epithelial cells (RPTEC) and of HK2, an immortalized proximal tubule epithelial cell line. rhIL-15 treatment attenuates TGF-*β*1-induced EMT of both RPTEC and HK2 cells interfering on TGF-*β*1 signaling.

These data suggest that (i) intrarenal IL-15 could act as a natural inhibitor of TGF-*β*1 and (ii) exogenous IL-15 treatment could be beneficial in some human nephropathies such as diabetic nephropathy.

## 2. Methods

### 2.1. Public Transcriptome Datasets

#### 2.1.1. Human Kidney Transcriptome in Context of Transplantation

Transcriptome matrix (GSE1563) normalized with software MAS 5.0 was downloaded on Gene Expression Omnibus (GEO) database (https://www.ncbi.nlm.nih.gov/geo/) and annotated with GEO platform GPL8300 corresponding to the technology Affymetrix Human Genome U95 Version 2 Array. Kidney tissue from transplanted patients with no clinical evidence of rejection (n=10) was compared to kidney tissue from transplanted patients with renal dysfunctions (n=5) [30].

#### 2.1.2. Kidney Tubulointerstitium Transcriptome in Context of Human Nephropathies

Transcriptome matrix (GSE47184) normalized with RMA algorithm was downloaded on GEO database and annotated with GEO platform GPL14663 corresponding to the technology Affymetrix GeneChip Human Genome HG-U133A. Kidney tubulointerstitium from control (n=4) was compared to the same tissue from different nephropathies such as diabetic nephropathy (n=11), minimal change disease (n=10), thin membrane disease (n=6), focal and segmental glomerulosclerosis (n=10), hypertensive nephropathy (n=1), IgA nephropathy (n=1), and membranous glomerulonephritis (n=18) [31].

#### 2.1.3. Bioinformatics and Statistical Analyses

Transcriptome analyses were performed in R software environment version 3.4.3. Boxplots were drawn with ggplot2 graphical definition [32]. Statistical tests of Fisher one-way analysis of variance (ANOVA) and two sided Student's t-test were performed with an error of 5 percent.

### 2.2. Cytokines, Antibodies and Reagents

Recombinant human (rh) TGF-*β*1 and IL-15 were purchased from Miltenyi Biotec (GmbH, Germany). Antibodies (Abs) against E-cadherin (AF648), IL-15 (IC2471P), IL-15R*α* (FAB1471P), IL-15R*β* (FAB224P), IL-15R*γ* (MAB2842), and TGF-*β*1 (AB-100-NA) and the U0126 MEK specific inhibitor (1144) were obtained from Bio-Techne Ltd. (Lille, France) and Abs against N-cadherin (4061), p-c-Jun (3270), vimentin (sc-73260), and Snail1 (3895) from Cell Signaling Technology (Leiden, Netherlands). Ab against ZO-1 (18-7430) was purchased from Invitrogen (Carlsbad, CA) and Abs against tubulin (sc-5274), GAPDH (sc-47724), and *β*-actin-HRP (sc-47778) from Santa Cruz Biotechnology (Heidelberg, Germany). Ab against Collagen IV (Ab6586) was purchased from Abcam (Cambridge, UK). Appropriate HRP- or Alexa-Fluor488 or TRITC conjugated secondary antibodies were from Jackson Immunoresearch Laboratories Inc. (West Grove, PA, USA). The specific JNK inhibitor SP600125 and fluorescein diacetate (FDA, F7378) were purchased from Sigma-Aldrich (Saint-Quentin, Fallavier, France).

### 2.3. Primary Cells and Cell Lines

Primary human renal proximal tubular epithelial cells (RPTEC) (Lonza, Verviers, Belgium) were expanded in vitro following manufacturer's instructions. In some experiments, a spontaneous EMT is induced in 5 days culturing cells in REGM medium without additive and daily medium renewal as previously described [23]. Immortalized human proximal tubule epithelial cells (HK-2), from the American Type Culture Collection (ATCC, Manassas, VA, USA), were cultured in complete Keratinocyte Serum Free Medium (K-SFM) according to ATCC instructions and were used between passages 3 and 15. Prior to cytokines exposure, cells were cultured in minimum starvation media containing antibiotics. Cells were then incubated with rhTGF-*β*1, rhIL-15, or/and anti-TGF-*β*1 neutralizing antibodies at indicated concentrations, for varying times.

### 2.4. Human Kidney Specimens

Paraffin-embedded sections from human renal biopsies were retrospectively analyzed. Informed written consent was given by the patients to use part of the biopsy for scientific purposes. All procedures and the use of tissues were performed in accordance with the Declaration of Helsinki principles.

### 2.5. TGF-*β*1 Quantification

Quantification of TGF-*β*1 in 2-5-day conditioned media from tubular cells was performed using a biological specific assay for active TGF-*β*1 [33] in which a TGF*β*-sensitive BL41 cell line was stably transfected with a reporter plasmid harboring a synthetic TGF-*β*-inducible DNA sequence upstream from the luciferase gene. The active form of TGF-*β*1 was quantified by directly processing the cell supernatant and the total TGF-*β*1 (i.e., active + latent) after acid activation of latent TGF-*β*1. Data were then normalized to the total cell number per well. The experiments were repeated 3 times in triplicate and are shown as means ± SEMs.

### 2.6. Collagen Quantification

The amount of collagen in the cell supernatant of 48h-treated cells was quantify using the commercially Sirius Red collagen detection kit (Chondrex, Inc., Redmond, USA) as per manufacturer's instructions. Briefly, collagen was first concentrated using the Concentrating Solution (catalog # 90626; Chondrex, Inc.) at 4°C for 24 hours. After dissolving the pellet in acetic acid, samples were incubated with Sirius Red solutions for 30 min at room temperature and eluted using an extraction buffer. The absorbance of the extracted solution was read at 540 nm by microplate reader. A calibration curve was constructed using bovine collagen-I in the range of 8-250*μ*g/mL.

### 2.7. Western Blotting

Western blot analysis was performed under reducing conditions from whole-cell lysates prepared in cold RIPA buffer as described previously [24]. Blots were subsequently probed overnight with indicated primary antibodies. Appropriate HRP-conjugated secondary antibodies were used. Densitometry of visualized bands was performed using Image J software (NIH).

### 2.8. Immunocytochemistry

Cells were dispensed into eight-well compartments of Lab-Tek tissue culture chamber slides (Nunc, Naperville, Ill.) and, at confluence, treated with the cytokines at indicated concentrations, for varying times. For membrane staining, cells were fixed with cold methanol:acetone (1:1) at -20°C for 10 min whereas a 4% paraformaldehyde fixation following by a 0.5% Triton X-100 permeabilization were performed for intracellular stainings. After a blocking step, cells were incubated at 4°C with the indicated primary and fluorochrome conjugated secondary antibodies diluted in blocking solution, between 3 washes. The cells were mounted in 4,6-diamidino-2-phenylindole (DAPI, Invitrogen,Cergy Pontoise, France) and visualized by fluorescence microscopy (Leica, Germany).

### 2.9. Immunohistochemistry

Paraffin-embedded kidney tissues were treated using the appropriate antigen-retrieval method, as previously described [34]. Tissues were stained with an anti-IL-15 and the Envision kit was applied for 45 min at room temperature (DakoCytomation, Denmark), according to the manufacturer's instructions. Staining was revealed using a DAB kit (DakoCytomation, Denmark) and counterstained with hematoxylin (Sigma-Aldrich, Saint-Louis, USA). Positive staining was quantified using computer-based morphogenic analysis software (TRIBVN ICS Framework) in a blinded manner. The entire kidney sagittal cross-section of each biopsy was selected for IL-15 quantification which represents around ~7mm^2^ of cortex surface area as described before [34]. The positive area in the cortex was measured for each specimen and expressed as a percentage of the total cortical kidney section.

### 2.10. Flow Cytometric Analysis

Cells were detached with Accutase (Sigma-Aldrich) and cell surface expression of IL-15 and IL-15R*α* was analyzed by flow cytometry as previously described [35]. Briefly, cells were washed in FACS buffer containing 1% FCS and 2 mM EDTA and stained with phycoerythrin- (PE-) conjugated antibodies directed against IL-15 and IL-15R*α*. After 3 washes, 10.000 cells were analyzed on a Fortessa flow cytometer (BD Biosciences) and the data was analyzed using FlowJo software (Tree Star Inc.). Three replicates were used for each condition and the experiment was repeated at least three times.

### 2.11. Statistical Analyses

We compared differences using the Mann-Whitney* U* test or the paired Student's t-test, as appropriate. All analyses were performed using GraphPad Prism 5.0 software. Data were considered statistically significant when the *p* value was <0.05. All data are expressed as means ± SEMs.

## 3. Results

### 3.1. IL-15 Expression Is Decreased in Transplants with Renal Dysfunction and Human Inflammatory Nephropathies

**Figure 1:**
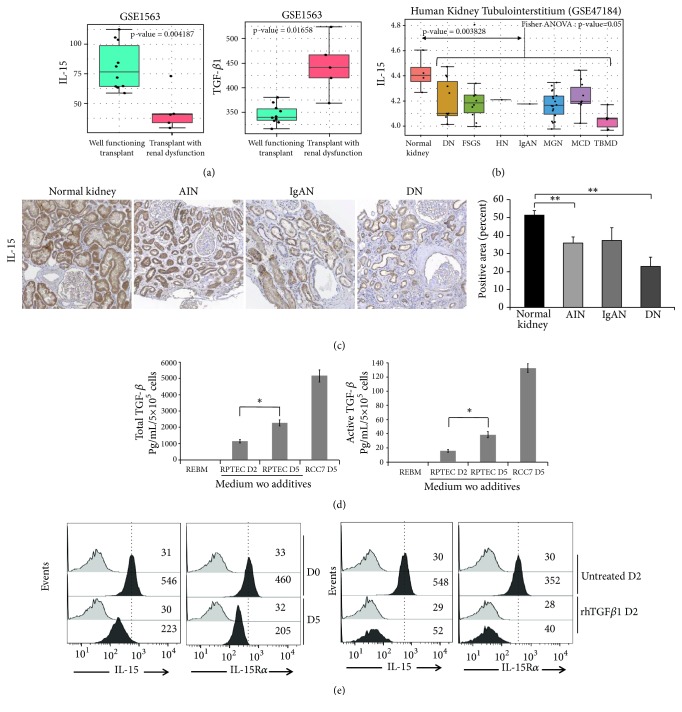
*IL-15/TGFβ ratio is unbalanced in human chronic kidney disease and in a “spontaneous EMT model”*. (a) Boxplot of IL-15 and TGF-*β*1 transcript quantification in transplant patients from well-functioning transplants with no clinical evidence of rejection in comparison of transplant patients from transplants with renal dysfunction without rejection (GSE1563); p values for IL-15 (*p*<0.01) and TGF-*β*1 (*p*<0.05) were estimated by two sided Student's t-test. (b) Boxplot of IL-15 mRNA transcript quantification by Affymetrix transcriptome (GSE47184) comparing kidney tubulointerstitium from divers human nephropathies (diabetic nephropathy (DN), focal segmental glomerulosclerosis (FSGS), hypertensive nephropathy (HN), IgA nephropathy (IgAN), membranous glomerulonephritis (MGN), Minimal Change Disease (MCD), thin basement membrane disease (TBMD) as compared to same tissue from normal kidney, p value from global comparison was performed with Fisher one-way ANOVA (*p*≤0.05), and comparison of control versus pooled nephropathies was done by two sided Student's t-test (*p*<0.01). (c) Immunohistochemistry for IL-15 in normal and pathological kidneys, including acute interstitial nephritis (AIN), IgAN, and DN (*n*=5–7 patients per group). Positive staining was quantified by morphometric analysis (bar chart). *∗∗p*<0.01. (d) In the “spontaneous EMT model” (five days of growth supplements deprivation and absence of daily medium) both total and active TGF-*β*1 forms were quantified in 2-5 days RPTEC-derived conditioned media using a biological specific assay (*∗* * p*<0.05, n=3, ±SEMs). Five days of conditioned media from the human renal cell carcinoma cell line RCC7 were used as positive control of TGF-*β*1 secretion. (e) Membrane-bound IL-15 and IL-15R*α* expression on RPTEC cells was analyzed by flow cytometric analysis after a 5 days “spontaneous EMT” (upper panels) or a 2-days rhTGF-*β*1 treatment (3 ng/mL, lower panels). Grey histograms refer to isotype-matched control and black histograms to surface IL-15 or IL-15R*α* molecules. Mean fluorescence intensity values for each marker are shown in each histogram. The data are representative of 3 separate experiments.

Since intrarenal IL-15 is strongly decreased in several experimental murine nephropathies compromising renal function [20, 21], we have first investigated the IL-15 expression profile for kidney biopsies of transplant patients and of human nephropathies by bioinformatics based on microarray datasets. Transcriptome experiments investigating human nephropathy diversity was already processed with Affymetrix technology. Analysis of the published GES1563 dataset [30] shows a significant downregulation of IL-15 transcript in transplants with renal dysfunction (n=5) versus well-functioning transplants with no clinical evidence of rejection (n=10) (Figure 1(a), left panel; t-test p value =0.0042). TGF-*β*1 was found inversely regulated in the same context of response to human transplantation and renal dysfunction (Figure 1(a), right panel; t-test p value=0.016). Kidney tubulointerstitium from patients with different nephropathies such as diabetic nephropathy, minimal change disease, thin membrane disease, focal and segmental glomerulosclerosis, hypertensive nephropathy, IgA nephropathy, and membranous glomerulonephritis was also compared to control tissue donors (GSE47184 dataset [31]). IL-15 transcript quantification in this transcriptome dataset revealed a significant regulation between analyzed group of samples (one-way ANOVA p value=0.05, Figure 1(b)). Boxplot performed on IL-15 transcript quantification showed a downregulation in several nephropathies as compared to the control donors and this global tendency was found confirmed by Student's t-test (p value = 0.0038). To confirm at protein level that IL-15 expression is reduced in human inflammatory nephropathies, immunohistochemistry was performed on paraffin-embedded kidney tissues from human renal biopsies. Indeed, immunohistochemistry analysis (Figure 1(c)) shows a high expression level of IL-15 in normal kidneys, mostly in tubular cells (n=5) while a significantly decreased IL-15 expression (p<0.01) was observed in acute interstitial nephritis (AIN) (n=5), IgA nephropathies (n=7), and diabetic nephropathy (n=6).

### 3.2. IL-15/TGF*β* Ratio Is Unbalanced in a “Spontaneous” EMT Model

We have recently shown that primary renal epithelial culture cells (RPTEC) deprived of growth supplements undergo within 5 days a “spontaneous” EMT process, which is inhibited treating cells with 10 pg/mL of rhIL-15 suggesting a renoprotective potential for this cytokine able to preserve epithelial phenotype [23, 35]. Since intrarenal TGF-*β* activation is a major inducer of EMT process [17], we investigated in our* in vitro* spontaneous EMT model, a potential TGF-*β* involvement. Using a biological specific assay for active TGF-*β*1 [33], we measured TGF-*β*1 concentration in conditioned medium of tubular cells cultured for 2 and 5 days in absence of growth supplements. In our tubular primary cultures, we observed a significant upregulation of the secretion of both active and total forms of TGF-*β*1 (Figure 1(d)).

Apart from the secreted form, an IL-15 form, anchored through the high affinity IL-15R*α* chain, was detected on cell surface of RPTEC cells. This membrane-bound IL-15 (mbIL-15), delivering the signal both* in cis* [36] and* in trans* [20] through the intermediate affinity receptor IL-15R*βγ*, is considered to be the dominant form of the intrarenal cytokine [20, 23]. Flow cytometric analysis after 5 days of deprivation of growth supplements shows that mbIL-15 and cell surface expression of IL-15R*α* are decreased along the “spontaneous” EMT process (Figure 1(e), upper panels), whereas those of IL-15R*β* and IL-15R*γ* are unaffected (supplementary Figure S1). To evaluate TGF-*β* involvement in this process, RPTEC cells were treated with recombinant human TGF-*β*1 (rhTGF-*β*1) in order to induce an EMT process. A concomitantly decreased expression of mbIL-15 and surface IL-15R*α* was observed on 2 days rhTGF-*β*1-treated RPTEC cells (Figure 1(e), lower panels), demonstrating that the IL-15/TGF*β* ratio is unbalanced in both* in vitro* tubular EMT models.

### 3.3. Both TGF-*β* Neutralization and rhIL-15 Treatment Inhibit the “Spontaneous EMT”

**Figure 2:**
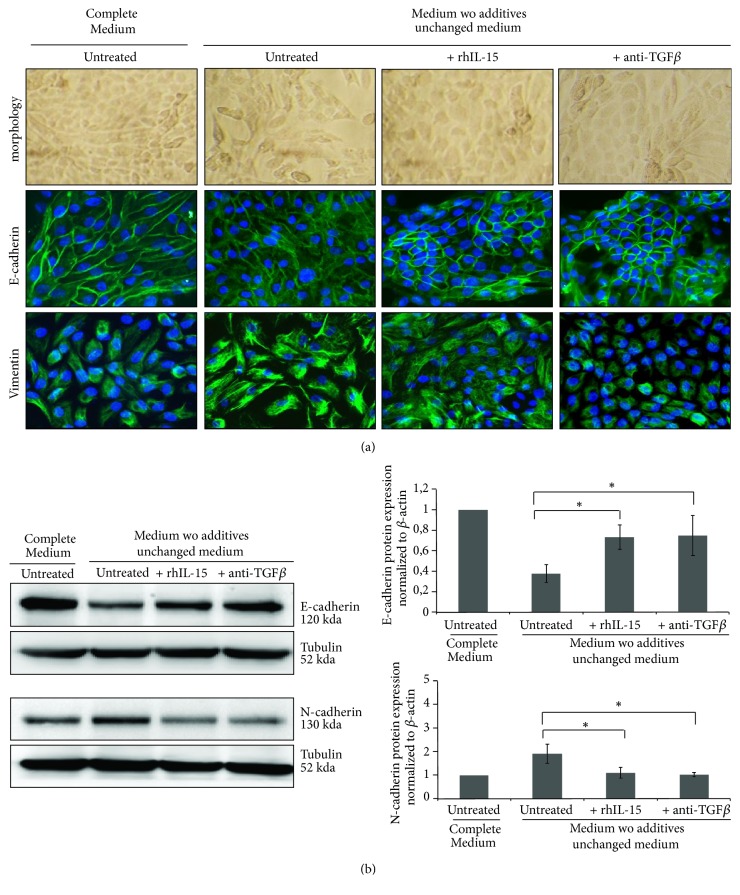
*Both TGF-β neutralization and rhIL-15 treatment inhibit the “spontaneous EMT”*. (a) Immunofluorescent staining of E-cadherin (epithelial marker) and vimentin (mesenchymal marker) expressions at day 5 in RPTEC cells under standard (complete REBM) and “spontaneous EMT” conditions, in presence or absence of neutralizing TGF-*β*1 antibody (5 *μ*g/mL) and/or rhIL-15 treatment (1 ng/mL). (b) E-cadherin (epithelial marker) and N-cadherin (mesenchymal marker) expressions were analyzed by Western blot at day 5 in RPTEC cells, under the same culture cell conditions and treatments. Bar charts represents E-cadherin and N-cadherin expression normalized to tubulin (n=3).

To prove TGF-*β* involvement in the “spontaneous” EMT process, characterized by the loss of the cobblestone morphology, a decreased E-cadherin expression associated with an increase of mesenchymal markers (vimentin and N-cadherin), tubular cells were incubated for 5 days with neutralizing anti-TGF-*β*1 mAbs. Similarly to rhIL-15 treatment, TGF-*β*1 neutralization preserves epithelial traits, with maintenance of E-cadherin expression (Figures 2(a) and 2(b)), preventing the upregulation of vimentin (Figure 2(a), supplementary Figure S2a) and N-cadherin (Figure 2(b)) expression. These results strengthen a possible interaction between these two cytokines in the regulation mechanisms of EMT as already observed in other process [25–29].

### 3.4. IL-15 Inhibits TGF-*β*1 Induced EMT in RPTEC and HK2 Cells

**Figure 3:**
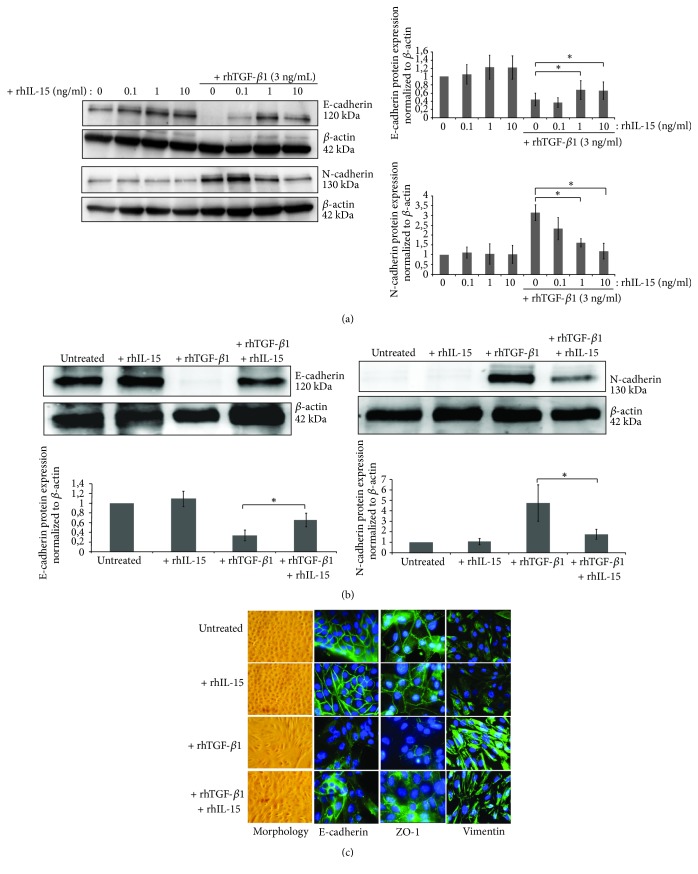
*rhTGF-β1-induced EMT in RPTEC and HK-2 cells is inhibited by in vitro rhIL-15 treatment*. (a) Analysis of E-cadherin and N-cadherin expressions in 48h-treated HK-2 cells by Western blotting using increasing concentrations of rhIL-15 (0.1-10 ng/mL) ± 3 ng/mL of rhTGF-*β*1. (*∗* * p*<0.05, n=4, ±SEMs). (b) The same experiment was realized on RPTEC cells using 1 ng/mL of rhIL-15 and 3 ng/mL of rhTGF-*β*1 for 48h. Bar charts represent E-cadherin and N-cadherin expression normalized to *β*-actin (*∗p*<0.05, n=4, ±SEMs). (c) Fluorescent immunostaining for the epithelial markers E-cadherin and ZO-1 and the mesenchymal marker vimentin, under “spontaneous EMT” culture conditions. Cells were treated for 48h with rhTGF-*β*1 (3 ng/mL) ± rhIL-15 (1 ng/mL). In left panels, cells were viewed using phase contrast microscopy. Original magnification ×63. These data are representative of three independent experiments.

We then investigated in both primary (RPTEC) and immortalized (HK-2) human proximal tubular epithelial cells, whether rhIL-15 could directly inhibit rhTGF-*β*-induced EMT. To examine the prospective efficacy of IL-15 in inhibiting TGF-*β*1-induced EMT, we first treated HK2 cells with increasing concentrations of rhIL-15 (0.1, 1, and 10 ng/mL) and we determined that 1 ng/mL was the rhIL-15 concentration able to inhibit more efficiently the downregulation of the epithelial marker E-Cadherin associated with the upregulation of the mesenchymal markers N-Cadherin (Figure 3(a)) and vimentin (supplementary Figure S2b) triggered by rhTGF-*β*1 treatment (3 ng/mL, 48h). The above-mentioned concentrations were subsequently employed in the following experiments. Similar results were observed in RPTEC cells (Figure 3(b)). These results were confirmed by immunofluorescent analysis on RPTEC cells (Figure 3(c)). Indeed, rhTGF-*β*1 triggers EMT process characterized by an alteration in cell morphology from the characteristic organized ‘cobblestone' appearance of differentiated epithelial cell monolayers to a disorganized elongated fibroblast-like phenotype, the disappearance of epithelial markers (E-cadherin and ZO-1), and the marked increase of the mesenchymal marker vimentin. Remarkably, rhIL-15 (1 ng/mL) coincubation efficiently counteracts TGF-*β*1 (3 ng/mL) action preserving the morphology change and epithelial traits of RPTEC cells.

### 3.5. IL-15 Inhibits TGF-*β*1 Induced Collagen IV Synthesis in RPTEC and HK2 Cells

**Figure 4:**
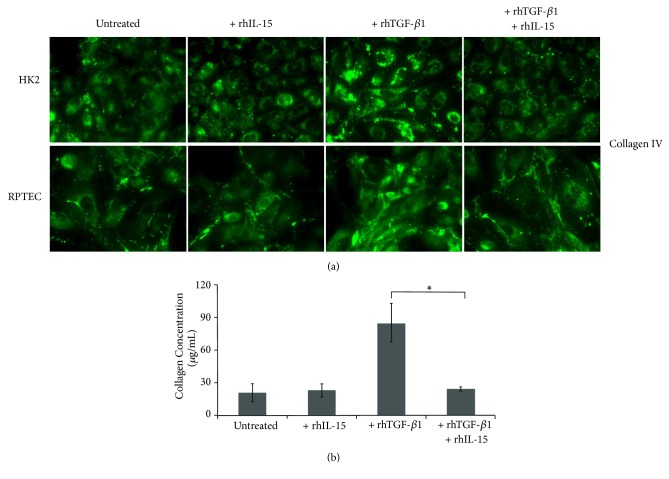
*rhIL-15 attenuated collagen synthesis and secretion induced by rhTGF-β1 stimulation in both RPTEC and HK-2 cells*. (a) Immunofluorescent staining of collagen IV expression in RPTEC and HK-2 cells treated for 48h with rhTGF-*β*1 alone (3 ng/mL), rhIL-15 alone (1 ng/mL), or both cytokines. Original magnification ×63. Data is representative of three independent experiments. (b) The amount of collagen in 48h-treated HK-2 cell supernatants was quantify using the commercially Sirius Red collagen detection kit. Data are mean ±SEMs.

Tubulointerstitial fibrosis in kidneys is characterized by the increased accumulation of ECM components largely due to their excessive production in parallel with their reduced degradation [37]. Subsequently, we investigated whether IL-15 could inhibit the terminal step of tubular EMT process analyzing its effect on collagen IV synthesis and secretion induced by rhTGF-*β*1 treatment. In Figure 4(a), immunofluorescent studies reveal that rhTGF-*β*1 treatment strongly increase within 24 hours collagen IV expression in secretive granules in both epithelial cell types, and such production was markedly suppressed by rhIL-15 treatment. This IL-15 inhibiting activity on collagen production was quantified using the Sirius Red Total Collagen Detection Kit (Figure 4(b)). Thus, 48h-rhTGF-*β*1 treatment induced a threefold increase of the collagens levels in HK2 cell supernatant, while rhIL-15 coincubation totally inhibited the stimulatory action of rhTGF-*β*1. Taken together, our results support the notion that rhIL-15 inhibits EMT induced by rhTGF-*β*1 in renal human tubular epithelial cells.

### 3.6. IL-15 Inhibits Snail1 Expression in TGF-*β*1-Stimulated HK-2 Cells through c-Jun Activation

**Figure 5:**
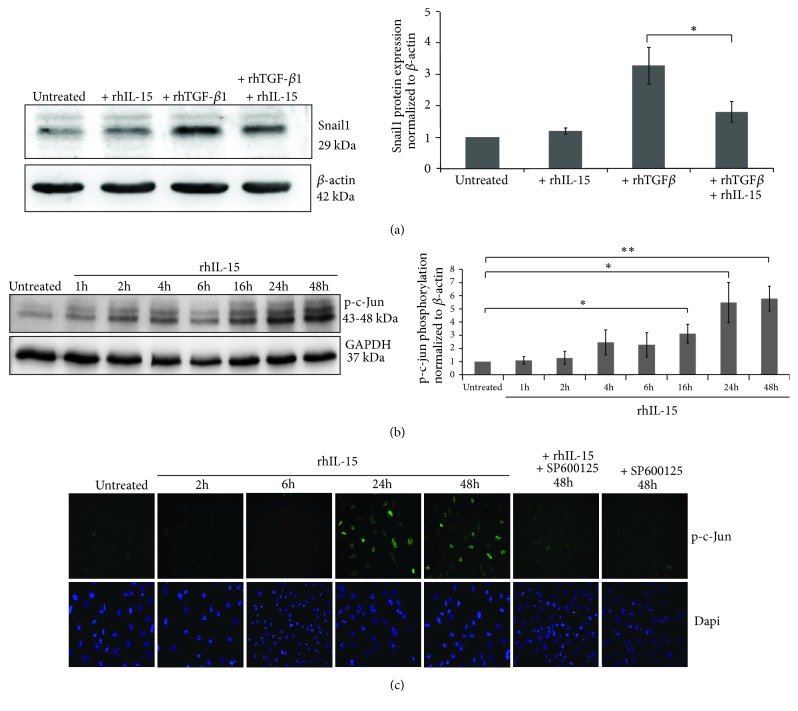
*IL-15 inhibited TGF-β-induced Snail1 expression in HK-2 cells through phospho-C-Jun upregulation*. (a) Western blot analysis of Snail1 expression after a 48h-rhTGF-*β*1 stimulation (3 ng/mL) in presence or absence of rhIL-15 treatment (1 ng/mL). Bar chart represents Snail1 expression normalized to *β*-actin (*∗p*<0.05, n=6, ±SEMs). (b) Western blot analysis and (c) Immunofluorescent staining of phospho-c-Jun expressed after 1h to 48h of rhIL-15 treatment (1 ng/mL) ± the specific JNK inhibitor SP600125. Bar chart represents phospho-c-Jun expression normalized to GAPDH (*∗∗p*<0.01, *∗p*<0.05, n=3, ±SEMs). To visualize cells, cell nuclei were stained with DAPI (Lower panels). Immunofluorescence data are representative of three independent experiments.

In order to decipher the mechanisms involved in rhIL-15 inhibition of rhTGF-*β*1-induced EMT, we investigated which molecular pathways controlled by rhTGF-*β*1 were affected by rhIL-15 treatment. TGF-*β*1 induces EMT via the smad3-dependent induction of transcriptional repressors, such as Snail1; a zinc finger transcription factor which acts as a key regulator of EMT, sufficient alone to transcriptionally repress E-cadherin and to induce EMT [16, 38]. Western blot analysis showed that the strongly rhTGF-*β*1-induced Snail1 expression is almost totally inhibited when cotreating HK-2 cells with rhIL-15 for 48h (Figure 5(a)). Subsequently, we investigated how rhIL-15 interferes on rhTGF-*β*1 signal transduction. Recent data in other cell models have shown that IL-15 does not inhibit the initial steps of Smad2/3 signaling pathway but acts on the formation of Smad2/3–DNA complexes, through the phospho-c-jun Smad3 corepressor [25]. Herein, in supplementary Figure S3, we confirm that rhIL-15 treatment does not inhibit TGF*β*R expression (TGF-*β*RI and TGF-*β*RII, Figure S3a) phosphorylation (Figure S3b) and nuclear translocation of Smad2/3 complex (Figure S3c) in rhTGF-*β*1-stimulated HK-2 cells.

We thus assessed whether the c-Jun pathway is involved in the repression of Snail1 expression analyzing c-Jun phosphorylation level in rhIL-15-treated HK2 cells. Western blotting (Figure 5(b)) and immunofluorescence (Figure 5(c)) studies revealed that rhIL-15 induced in HK-2 cells a marked increase of p-c-Jun expression only after a prolonged cytokine stimulation (24h-48h). This effect was blocked when coincubating the cells with the SP600125 molecule, a selective inhibitor of c-Jun N-terminal kinase (JNK) (Figure 5(c), supplementary Figure S4).

**Figure 6:**
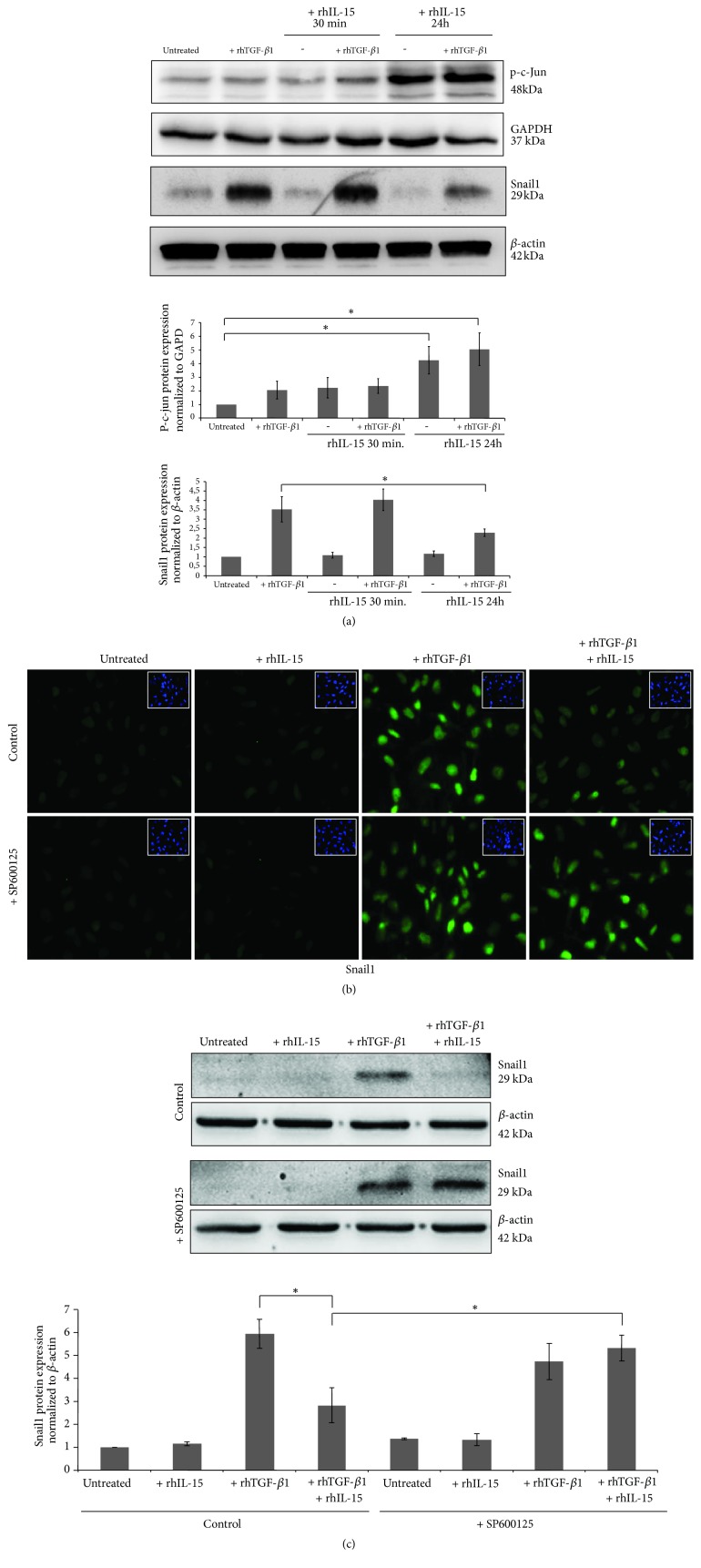
*Inhibition of rhTGF-β1-induced Snail1 expression by rhIL-15 involved the activation of C-jun pathway*. (a) C-Jun phosphorylation and Snail1 expression were analyzed by western blotting after a 6h rhTGF-*β*1 treatment (3 ng/mL) in HK-2 cells pretreated or not with rhIL-15 for 30 min or 24h of (1 ng/mL). Bar charts represent phospho-c-Jun and Snail1 expressions normalized to GAPDH and *β*-actin, respectively. (*∗p*<0.05, n=3, ±SEMs). Snail1 expression analyzed by fluorescent immunostaining (b) or western blotting (c) after a 6h rhTGF-*β*1 (3 ng/mL) treatment in HK-2 cells pretreated or not for 24h with rhIL-15 (1 ng/mL). In some conditions, the specific JNK inhibitor SP600125 was added. To visualize cells, cell nuclei were stained with DAPI (insets). Original magnification ×25. Immunofluorescence data are representative of three independent experiments. Bar chart represents Snail1 expression normalized to *β*-actin, respectively (*∗p*<0.05, n=3, ±SEMs).

To prove the specific involvement of p-c-Jun in the repression of Snail1 expression in rhIL-15/rhTGF-*β*1 cotreated cells, HK2 cells were pretreated with rhIL-15 for 30 min or 24h before inducing Snail1 expression under a 6h rhTGF*β*-1 treatment. rhTGF-*β* treatment alone did not activate c-Jun and did not interfere on the c-Jun phosphorylation which is only induced after long-term rhIL-15 incubation (Figure 6(a), upper panel). Interestingly, only this latter condition inhibits TGF-*β*1-induced Snail1 expression (Figure 6(a), lower panel). Immunofluorescence analysis (Figure 6(b)) and western blotting (Figure 6(c)) strengthen these data since the rhIL-15 inhibitory activity on TGF-*β*1-induced Snail1 nuclear translocation was blocked by the specific inhibitor of JNK SP600125, showing that Snail1 inhibition by rhIL-15 depends on c-Jun activation.

## 4. Discussion

Renal tubulointerstitial fibrosis, characterized by accumulation of myofibroblasts responsible for the excessive matrix deposition, is the final common outcome in almost all progressive chronic kidney diseases (CDK) [8]. In this context, the exact source of these cells is unclear and controversial and only in few nephropathies such as diabetic nephropathy, the development of renal fibrosis is more clearly associated with intrarenal TGF-*β*1 induced transition of secondary epithelial cells towards myofibroblastic cells: the so-called Type II EMT [13–15]. Interestingly, a recent study shed a new light on the role of the EMT in renal fibrosis. Indeed, Grande et al. demonstrate that* in vivo* a mechanism of partial and reversible tubular EMT, induced by Snail1 reactivation, relays to the interstitium signals that promote myofibroblast differentiation from renal and bone marrow-derived cells, fibrogenesis, and sustain inflammation [16].

The molecular mechanisms involved in EMT are very complex and a number of signaling networks and mediators have been identified in regulating this process. Among the fibrotic factors, TGF-*β*, whose upregulation have been linked to fibrosis development in DN, membranous nephropathy and other CDK, is a major inducer of EMT and a central mediator of renal fibrosis [4]. Several endogenous antifibrotic factors, particularly HGF and BMP-7, which can precisely antagonize the fibrogenic action of TGF-*β*, have also been identified [39–41]. Moreover, the above-mentioned factors are downregulated in chronic kidney injury [4, 19], showing that the imbalance of fibrotic/antifibrotic factors is a central parameter of tissue fibrosis. An important strategy for antifibrotic therapy would be to increase or restore the expression of antifibrotic factors in the diseased kidney [4]. However, only few clinical trials have successfully targeted fibrosis in CKD, likely reflecting the fact that inflammation is multifactorial and self-sustaining. Thus, it is necessary to consider others antifibrotic factors that could accomplish this job.

In this context, a large body of evidence supports the hypothesis that Interleukin-15 (IL-15), whose functions have been underestimated in human renal physiopathology, could be one of these candidates. IL-15 is a pleiotropic cytokine involved not only in innate immunity, but also in functions outside the immune system. Notably, renal tubular and cortical epithelial cells are an important source of IL-15 which acts under several forms. Indeed, renal cells secrete at low levels the monomeric IL-15 which can act in an auto/paracrine manner through the high affinity IL-15R*αβγ* complex, behaving as a powerful survival factor increasing in particular Bcl-2/Bax ratio [20, 21, 42]. Moreover, these cells also express a membrane-bound IL-15 form, anchored to the cell surface through the IL-15R*α* chain (mbIL-15) [35], whose transpresentation to neighboring cells expressing the intermediate affinity receptor (IL-15R*βγ*) is considered in the murine model as a major physiologic mechanism assuring renal homeostasis [20].

Several studies have shown that TGF-*β* and IL-15 display opposite actions. Thus, IL-15 is a prominent antiapoptotic factor for primary cultured RPTEC, while TGF*β* displays a proapoptotic function [20, 43]. Moreover, IL-15 counteracts TGF-*β*1-induced myofibroblast differentiation in human fetal lung fibroblasts and could exert antifibrotic effects in certain forms of pulmonary fibrosis [26]. By contrast in celiac disease, IL-15 sustains intestinal inflammation, inhibiting TGF-*β*-mediated immunosuppressive signaling in human T lymphocytes [25]. Finally, IL-15 is able to counteract immunosuppressive effects exerted by TGF-*β* on NK cells from AML patients [27].

On the basis of the above-mentioned results, we propose that intrarenal IL-15 could act as an endogenous regulator of TGF-*β*1 activity. To confirm this hypothesis, we first investigated IL-15 expression in various human nephropathies. Interestingly, we found a significant IL-15 decreased expression at transcriptional level in human nephropathies by bioinformatics based on microarray datasets. This reduced IL-15 expression was validated at protein level by immunohistochemistry in interstitial nephritis and DN, whereas IL-15 expression in normal kidneys is high, mostly in tubules as previously described in experimental nephropathies [20, 21]. At the same time, we confirmed that RPTEC cells starved of growth supplements rapidly undergo spontaneous EMT that is efficiently blocked not only by rhIL-15 treatment as previously shown [23] but also using a neutralizing anti-TGF-*β*1 mAbs. In addition, we show that, in this model, RPTEC cells rapidly exhibit a significant decrease of membrane-bound IL-15 and IL-15R*α* expression, whereas those of IL-15R*β* and IL-15R*γ* are not affected. Concomitantly, TGF-*β*1 secretion is increased, reproducing what observed in experimental inflammatory nephropathies. The fact that rhTGF-*β*1 treatment causes a rapid decrease of the surface expression of IL-15 and IL-15R*α* on RPTEC cells suggest that this phenomenon is associated both* in vitro* and* in vivo* to the activation of intra-renal TGF-*β*1. Thus, it is possible that, in some human nephropathies, an unbalanced production of intrarenal IL-15 and TGF-*β*1 could render epithelial cells more prone to fibrogenic stimuli.

**Figure 7:**
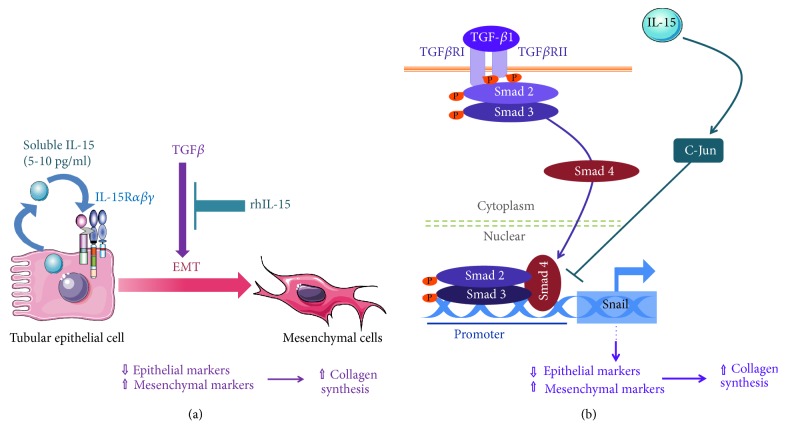
*Recapitulative diagram of IL-15 pathway involved in TGF-β signaling*. (a) rhIL-15 treatment counteract EMT in renal epithelial cells, preserving epithelial markers (E-cadherin and ZO-1) and inhibiting the expression of mesenchymal ones (N-cadherin, vimentin) and the production/secretion of collagen IV. (b) Dissecting the IL-15 inhibitory mechanisms reveal that the early steps of TGF-*β*-mediated signaling (phosphorylation and nuclear translocation of Smad2/3 complex) are not impaired by rhIL-15. However, rhIL-15 interferes on the induction of the transcription factor Snail1, a master regulator of EMT, through a long-lasting activation of c-jun pathway, consistent with the demonstrated inhibitory effect of phospho-c-jun on the formation of Smad2/3-DNA complexes.

To demonstrate the role of IL-15 in protecting human renal epithelial cells from TGF-*β*1-induced EMT* in vitro*, we used RPTEC (primary cells) and HK-2 cells (immortalized cell line), two epithelial models widely used to study EMT. First results show that rhIL-15 treatment counteracts EMT in both cell types, preserving epithelial markers (E-cadherin and ZO-1) and inhibiting the expression of mesenchymal ones (N-cadherin, vimentin) and the production/secretion of total collagen (i.e., collagen IV) (Figure 7(a)). We determined that 1 ng/mL is the minimal rhIL-15 concentration able to inhibit TGF-*β*1 effects indicating that IL-15 action is mediated by the intermediate affinity receptor IL-15R*βγ* on these cells. Dissecting the IL-15 inhibitory mechanisms reveal that rhIL-15 interferes with the induction of the transcription factor Snail, a master regulator of EMT, through a long-lasting activation of c-jun pathway (Figure 7(b)). Blocking Snail1 activation by IL-15 is a crucial step since it is likely that intrarenal IL-15 could blunt the partial tubular EMT triggered by Snail which* in vivo* represents an essential check point promoting myofibroblast differentiation from different types of renal and bone marrow-derived cells and fibrogenesis [16]. Furthermore, rhIL-15 treatment at 1 ng/mL efficiently counteracts rhTGF-*β*1-induced apoptosis, through the MAPK pathway depending on the IL-15R*βγ* complex (supplementary Figure S5), demonstrating that the survival factor IL-15 can interfere directly with tubular TGF-*β*-induced apoptosis which correlates with tubulointerstitial fibrosis in experimental nephropathies [21].

The process of renal fibrosis, in which multiple cellular events and molecular mediators participate and interact in concert, is enormously complicated. In this context, intrarenal IL-15 could act on other events involved in the renal fibrosis, inhibiting for instance the induction of the nephritogenic chemokine, MCP-1, a powerful chemoattractant for macrophages that are a powerful source of TGF-*β* [21]. Alternatively IL-15 could counteract TGF-*β*1-induced fibroblast-to-myofibroblast differentiation as reported in a pulmonary fibrosis model [26]. Thus, acting on different actors of renal fibrosis, in particular tubular cells, it is tenting to speculate that the decreased IL-15 activity during kidney injury could be a crucial event in the fibrosis development. Moreover, IL-15 expression is reduced in biopsies of transplanted patients with renal dysfunction without rejection in comparison of transplant patients from well-functioning transplants without rejection. Taken together, these data suggest that a decline in renal-derived IL-15 is detrimental to kidney function, favouring renal fibrosis development.

Interestingly, decrease in intrarenal IL-15 was closely linked to changes in IL-15R*α* in several murine experimental nephropathies [20, 21]. Thus, the downregulation of intrarenal IL-15R*α* chain could be a major limiting factor impairing not only the action of the secreted monomeric cytokine through the decreased availability of the high affinity receptor but also the formation of the membrane-bound IL-15 on epithelial cells.

Therefore, restoration of the balance between pro- and antifibrotic signaling in the diseased kidney appear to be an important therapeutic strategy for preventing or delaying (or even reversing) renal fibrosis associated with CKD. In this regard, targeting TGF-*β*1 and its signaling pathway is only half the equation. Another approach could be to increase the antifibrotic factors, such as IL-15, in the diseased kidney.

In conclusion, IL-15 is an important homeostatic factor for tubular epithelial cells, able to counteract TGF-*β* induced apoptosis and EMT in both primary (RPTEC) and immortalized (HK-2) human proximal tubular epithelial cells. In this context, an increase of TGF-*β* production associated with a decreased activity of endogenous antifibrotic factors such as IL-15 may explain, at least in part, fibrosis development and renal failure in multiple nephropathies. It is tempting to suggest that intrarenal mbIL-15 is a natural inhibitor of intrarenal TGF-*β*1 and that therapeutic strategy to restore or increase IL-15 level in injured kidney could be beneficial in some human nephropathies such as diabetic nephropathy.

## Data Availability

The data used to support the findings of this study are included within the article and supplementary data.

## Supplementary Materials

Supplementary Materials Supplementary Figure S1.* Expression of IL-15Rβ and IL-15Rγ chains are unaffected along the spontaneous EMT process*. Cell surface expression of the IL-15R*β* and IL-15R*γ* chains on RPTEC cells was analyzed by flow cytometric analysis after 5 days in the “spontaneous” EMT model. Grey histograms refer to isotype-matched control and black histograms to the expression of IL-15R chains. Mean fluorescence intensity values for each marker are shown in each histogram. Supplementary Figure S2.* Inhibition of rhTGF-β1-induced vimentin expression by rhIL-15.* (**a**) Vimentin (mesenchymal marker) expression was analyzed by Western blot at day 5 in RPTEC cells under standard (complete REBM) and “spontaneous EMT” conditions, in presence or absence of neutralizing TGF-*β*1 antibody (5 *μ*g/mL) and/or rhIL-15 treatment (1 ng/mL) (n=1). (**b**) Analysis of vimentin expression by western blotting on RPTEC cells using 1 ng/mL of rhIL-15 and 3 ng/mL of rhTGF-*β*1 for 48h (n=1). Supplementary Figure S3.* rhIL-15 treatment did not affect TGF-βR expression, nor Smad2 and Smad3 phosphorylation and nuclear translocation in rhTGFβ1-treated HK-2 cells*. (**a**) Western blot analysis of TGF-*β*RI and TGF-*β*RII after 24h or 48h rhIL-15 treatment (1 ng/mL). Bar charts represent TGF-*β*RI and TGF-*β*RII expression normalized to GAPDH (n=3, ±SEMs). Antibodies (Abs) against TGF*β*RI (AF3025) and TGF*β*RII (AF-241-NA) were obtained from R&D Systems Europe Ltd., Abingdon, UK. (**b**) Smad2/3 expression and phosphorylation were analyzed by western blotting after a rhTGF*β*1 treatment (3 ng/mL, 30 min) in HK-2 cells pretreated or not with rhIL-15 (1 ng/mL, for 24h). Bar charts represent p-Smad2 and p-Smad3 expression normalized to their native form (n=3, ±SEMs). GAPDH is shown as a loading control. Abs against P-Smad2 (400800), Smad2 (511300), P-Smad3 (44246G), and Smad3 (511500) were purchased from Invitrogen (Carlsbad, CA). (**c**) Smad2 nuclear translocation (SAB4300562, Sigma-Aldrich) was revealed by immunofluorescent staining under the same cytokine treatments. Immunofluorescence data are representative of three independent experiments. Supplementary Figure S4.* Phosphorylation of C-Jun induced by rhIL-15 is inhibited by SP600125 inhibitor*. Western blot analysis of phospho-c-Jun expressed after 16h to 48h of rhIL-15 treatment (1 ng/mL) ± the specific JNK inhibitor SP600125. Phospho-c-Jun expression normalized to *β*-actin. Supplementary Figure S5.* rhIL-15 inhibits apoptosis induced by rhTGF-β1 in RPTEC cells*. RPTEC cells were treated with or without U0126 (10 *μ*M) and rhIL-15 (1 ng/mL) in the presence of TGF-*β*1 (3 ng/ml) for 48 h. After rhIL-15 and/or TGF-*β*1 treatments, cells were incubated for 5 min at 37°C with the fluorescent probe fluorescein diacetate (FDA) at 0.2 *μ*g/ml to assess cell viability (FDA-green positive, alive cells). 10.000 cells were analyzed on a Fortessa flow cytometer (BD Biosciences). rhIL-15 treatment inhibited apoptosis induced by rhTGF-*β*1 in RPTEC cells whereas cotreatment with U0126, a specific inhibitor of the ERK1/2 upstream kinase MEK1/2, completely abrogated rhIL-15 activity. Bar chart represents the percentage of apoptotic cells (FDA-green negative cells) (*∗* * p*<0.05, n=3, ±SEMs).
