# Correction to “Inhibition of TGF‐*β*1 Signaling by IL‐15: A Novel Role for IL‐15 in the Control of Renal Epithelial‐Mesenchymal Transition: IL‐15 Counteracts TGF‐*β*1‐Induced EMT in Renal Fibrosis”

**DOI:** 10.1155/ijcb/9758548

**Published:** 2026-07-30

A. Devocelle, L. Lecru, H. François, C. Desterke, C. Gallerne, P. Eid, O. Estelle, B. Azzarone, and J. Giron‐Michel, “Inhibition of TGF‐*β*1 Signaling by IL‐15: A Novel Role for IL‐15 in the Control of Renal Epithelial‐Mesenchymal Transition: IL‐15 Counteracts TGF‐*β*1‐Induced EMT in Renal Fibrosis,” International Journal of Cell Biology 2019, no. 1 (2019): 1–15, 10.1155/2019/9151394.

In the article titled “Inhibition of TGF‐*β*1 Signaling by IL‐15: A Novel Role for IL‐15 in the Control of Renal Epithelial‐Mesenchymal Transition: IL‐15 Counteracts TGF‐*β*1‐Induced EMT in Renal Fibrosis,” there is an error in Figure 3c. The image of the cell morphology in the “Untreated” panel represented the cells in the “rhIL‐15” group, resulting in the two panels sharing overlapping features.

This error occurred during figure assembly and Figure 3c should be corrected as follows:

**Figure 3:**
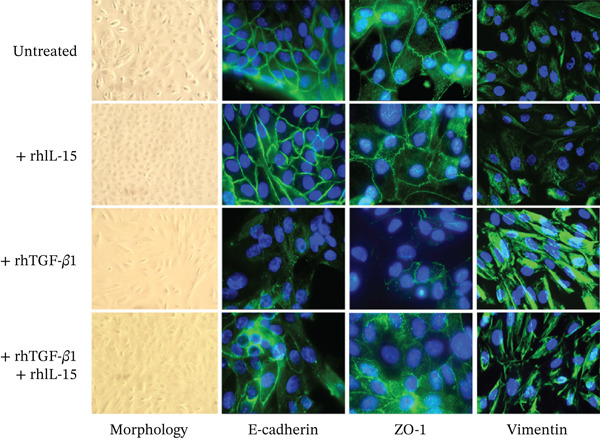
rhTGF‐*β*1‐induced EMT in RPTEC and HK‐2 cells is inhibited by in vitro rhIL‐15 treatment. (c) Fluorescent immunostaining for the epithelial markers E‐cadherin and ZO‐1 and the mesenchymal marker vimentin, under “spontaneous EMT” culture conditions. Cells were treated for 48 h with rhTGF‐*β*1 (3 ng/mL) ± rhIL − 15 (1 ng/mL). In left panels, cells were viewed using phase contrast microscopy. Original magnification: ×63. These data are representative of three independent experiments.

We apologize for this error.

