# Supplementary material for: Inhibition of TGF-β1 Signaling by IL-15: A Novel Role for IL-15 in the Control of Renal Epithelial-Mesenchymal Transition: IL-15 Counteracts TGF-β1-Induced EMT in Renal Fibrosis
Source: Int J Cell Biol. 2019 Jul 7;2019:9151394. doi: 10.1155/2019/9151394 (PMC6642769; doi:10.1155/2019/9151394)
Supplement: Supplementary Materials — Supplementary Figure S1. Expression of IL-15Rβ and IL-15Rγ chains are unaffected along the spontaneous EMT process. Cell surface expression of the IL-15Rβ and IL-15Rγ chains on RPTEC cells was analyzed by flow cytometric analysis after 5 days in the “spontaneous” EMT model. Grey histograms refer to isotype-matched control and black histograms to the expression of IL-15R chains. Mean fluorescence intensity values for each marker are shown in each histogram. Supplementary Figure S2. Inhibition of rhTGF-β1-induced vimentin expression by rhIL-15. (a) Vimentin (mesenchymal marker) expression was analyzed by Western blot at day 5 in RPTEC cells under standard (complete REBM) and “spontaneous EMT” conditions, in presence or absence of neutralizing TGF-β1 antibody (5 μg/mL) and/or rhIL-15 treatment (1 ng/mL) (n=1). (b) Analysis of vimentin expression by western blotting on RPTEC cells using 1 ng/mL of rhIL-15 and 3 ng/mL of rhTGF-β1 for 48h (n=1). Supplementary Figure S3. rhIL-15 treatment did not affect TGF-βR expression, nor Smad2 and Smad3 phosphorylation and nuclear translocation in rhTGFβ1-treated HK-2 cells. (a) Western blot analysis of TGF-βRI and TGF-βRII after 24h or 48h rhIL-15 treatment (1 ng/mL). Bar charts represent TGF-βRI and TGF-βRII expression normalized to GAPDH (n=3, ±SEMs). Antibodies (Abs) against TGFβRI (AF3025) and TGFβRII (AF-241-NA) were obtained from R&D Systems Europe Ltd., Abingdon, UK. (b) Smad2/3 expression and phosphorylation were analyzed by western blotting after a rhTGFβ1 treatment (3 ng/mL, 30 min) in HK-2 cells pretreated or not with rhIL-15 (1 ng/mL, for 24h). Bar charts represent p-Smad2 and p-Smad3 expression normalized to their native form (n=3, ±SEMs). GAPDH is shown as a loading control. Abs against P-Smad2 (400800), Smad2 (511300), P-Smad3 (44246G), and Smad3 (511500) were purchased from Invitrogen (Carlsbad, CA). (c) Smad2 nuclear translocation (SAB4300562, Sigma-Aldrich) was revealed by immunofluorescent staining under the same c [file 9151394.f1.zip › 9151394_SupplDesc.docx]

***Supplementary figure S1.*** ***Expression of IL-15Rβ and IL-15Rγ chains are unaffected*** ***along the spontaneous EMT process.***

Cell surface expression of the IL-15Rβ and IL-15Rγ chains on RPTEC cells was analyzed by flow cytometric analysis after a 5 days in the "spontaneous” EMT model. Grey histograms refer to isotype-matched control and black histograms to the expression of IL-15R chains. Mean fluorescence intensity values for each marker are shown in each histogram.

***Supplementary figure S2. Inhibition of rhTGF-β1-induced vimentin expression by rhIL-15*** (**a**) Vimentin (mesenchymal marker) expression was analyzed by Western blot at day 5 in RPTEC cells under standard (complete REBM) and "spontaneous EMT" conditions, in presence or absence of neutralizing TGF-β1 antibody (5 µg/mL) and/or rhIL-15 treatment (1 ng/mL) (n=1). (**b**) Analysis of vimentin expression by Western blotting on RPTEC cells using 1 ng/mL of rhIL-15 and 3 ng/mL of rhTGF-β1 for 48h (n=1).

***Supplementary figure S3. rhIL-15 treatment did not affect TGF-βR expression, nor Smad2 and Smad3 phosphorylation and nuclear translocation in rhTGFβ1-treated HK-2 cells.***

(**a**) Western blot analysis of TGF-βRI and TGF-βRII after 24h or 48h rhIL-15 treatment (1 ng/mL). Bar charts represent TGF-βRI and TGF-βRII expression normalized to GAPDH (n=3, ±SEMs). Antibodies (Abs) against TGFβRI (AF3025) and TGFβRII (AF-241-NA) were obtained from (R&D Systems Europe Ltd, Abingdon, U.K). (**b**) Smad2/3 expression and phosphorylation were analyzed by Western blotting after a rhTGFβ1 treatment (3 ng/mL, 30 min) in HK-2 cells pretreated or not with rhIL-15 (1 ng/mL, for 24h). Bar charts represent p-Smad2 and p-Smad3 expression normalized to their native form (n=3, ±SEMs). GAPDH is showed as a loading control. Abs against P-Smad2 (400800), Smad2 (511300), P-Smad3 (44246G), and Smad3 (511500) were purchased from Invitrogen (Carlsbad, CA). (**c**) Smad2 nuclear translocation (SAB4300562, Sigma-Aldrich) was revealed by immunofluorescent staining under the same cytokine treatments. Immunofluorescence data are representative of three independent experiments.

***Supplementary figure S4. Phosphorylation of C-Jun induced by rhIL-15 is inhibited by SP600125 inhibitor.***

Western-blot analysis of phospho-c-Jun expressed after 16h to 48h of rhIL-15 treatment (1 ng/mL) ± the specific JNK inhibitor SP600125. Phospho-c-Jun expression normalized to β-actin.

***Supplementary figure S5.*** ***rhIL-15 inhibits apoptosis induced by rhTGF-β1 in RPTEC cells.***

RPTEC cells were treated with or without U0126 (10 μM) and rhIL-15 (1 ng/mL) in the presence of TGF-β1 (3 ng/ml) for 48 h. After rhIL-15 and/or TGF-β1 treatments, cells were incubated for 5 min at 37 °C with the fluorescent probe fluorescein diacetate (FDA) at 0.2 μg/ml to assess cell viability (FDA*-*green positive*,* alive cells). 10.000 cells were analyzed on a Fortessa flow cytometer (BD Biosciences). rhIL-15 treatment inhibited apoptosis induced by rhTGF-β1 in RPTEC cells whereas co-treatment with U0126, a specific inhibitor of the ERK1/2 upstream kinase MEK1/2, completely abrogated rhIL-15 activity. Bar chart represents the percentage of apoptotic cells (FDA*-*green negative cells) (* *p*<0.05, n=3, ±SEMs).
