# Supplementary figures and images for: Inhibition of TGF-β1 Signaling by IL-15: A Novel Role for IL-15 in the Control of Renal Epithelial-Mesenchymal Transition: IL-15 Counteracts TGF-β1-Induced EMT in Renal Fibrosis
Source: Int J Cell Biol. 2019 Jul 7;2019:9151394. doi: 10.1155/2019/9151394 (PMC6642769; doi:10.1155/2019/9151394)

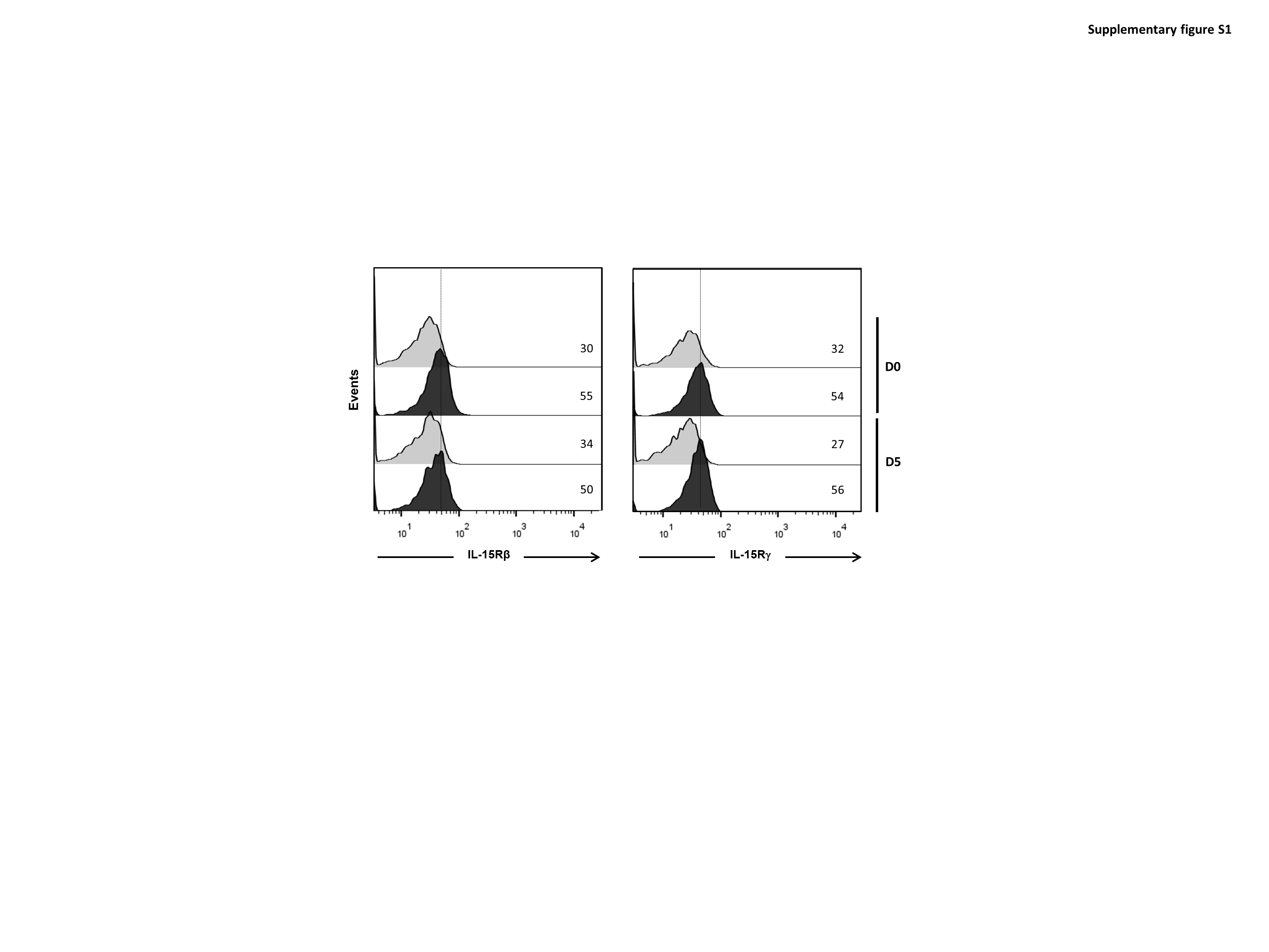

Supplement: Supplementary Materials — Supplementary Figure S1. Expression of IL-15Rβ and IL-15Rγ chains are unaffected along the spontaneous EMT process. Cell surface expression of the IL-15Rβ and IL-15Rγ chains on RPTEC cells was analyzed by flow cytometric analysis after 5 days in the “spontaneous” EMT model. Grey histograms refer to isotype-matched control and black histograms to the expression of IL-15R chains. Mean fluorescence intensity values for each marker are shown in each histogram. Supplementary Figure S2. Inhibition of rhTGF-β1-induced vimentin expression by rhIL-15. (a) Vimentin (mesenchymal marker) expression was analyzed by Western blot at day 5 in RPTEC cells under standard (complete REBM) and “spontaneous EMT” conditions, in presence or absence of neutralizing TGF-β1 antibody (5 μg/mL) and/or rhIL-15 treatment (1 ng/mL) (n=1). (b) Analysis of vimentin expression by western blotting on RPTEC cells using 1 ng/mL of rhIL-15 and 3 ng/mL of rhTGF-β1 for 48h (n=1). Supplementary Figure S3. rhIL-15 treatment did not affect TGF-βR expression, nor Smad2 and Smad3 phosphorylation and nuclear translocation in rhTGFβ1-treated HK-2 cells. (a) Western blot analysis of TGF-βRI and TGF-βRII after 24h or 48h rhIL-15 treatment (1 ng/mL). Bar charts represent TGF-βRI and TGF-βRII expression normalized to GAPDH (n=3, ±SEMs). Antibodies (Abs) against TGFβRI (AF3025) and TGFβRII (AF-241-NA) were obtained from R&D Systems Europe Ltd., Abingdon, UK. (b) Smad2/3 expression and phosphorylation were analyzed by western blotting after a rhTGFβ1 treatment (3 ng/mL, 30 min) in HK-2 cells pretreated or not with rhIL-15 (1 ng/mL, for 24h). Bar charts represent p-Smad2 and p-Smad3 expression normalized to their native form (n=3, ±SEMs). GAPDH is shown as a loading control. Abs against P-Smad2 (400800), Smad2 (511300), P-Smad3 (44246G), and Smad3 (511500) were purchased from Invitrogen (Carlsbad, CA). (c) Smad2 nuclear translocation (SAB4300562, Sigma-Aldrich) was revealed by immunofluorescent staining under the same c [file 9151394.f1.zip › Suppl Figure 1_IJCB_2781290.docx]

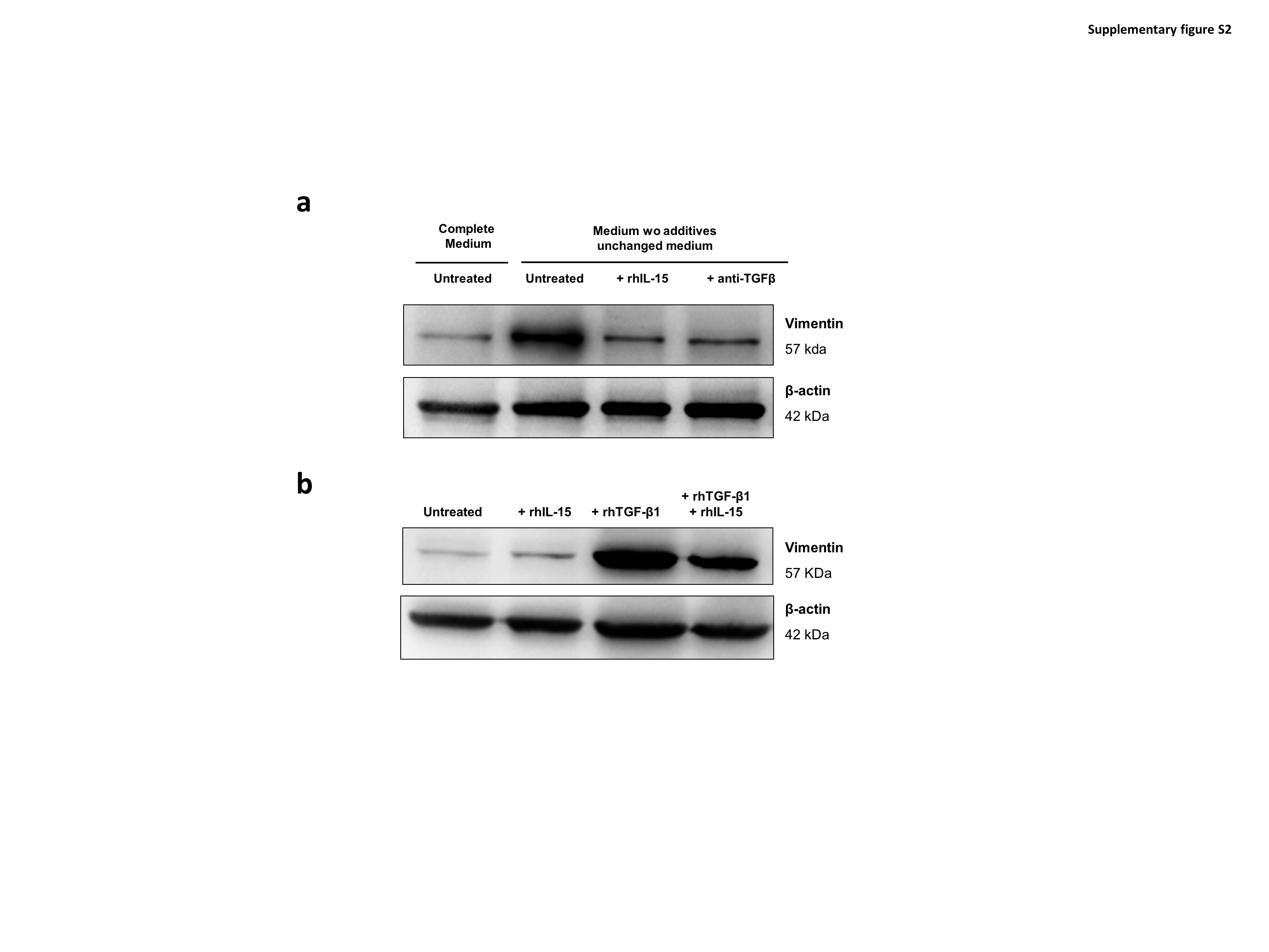

Supplement: Supplementary Materials — Supplementary Figure S1. Expression of IL-15Rβ and IL-15Rγ chains are unaffected along the spontaneous EMT process. Cell surface expression of the IL-15Rβ and IL-15Rγ chains on RPTEC cells was analyzed by flow cytometric analysis after 5 days in the “spontaneous” EMT model. Grey histograms refer to isotype-matched control and black histograms to the expression of IL-15R chains. Mean fluorescence intensity values for each marker are shown in each histogram. Supplementary Figure S2. Inhibition of rhTGF-β1-induced vimentin expression by rhIL-15. (a) Vimentin (mesenchymal marker) expression was analyzed by Western blot at day 5 in RPTEC cells under standard (complete REBM) and “spontaneous EMT” conditions, in presence or absence of neutralizing TGF-β1 antibody (5 μg/mL) and/or rhIL-15 treatment (1 ng/mL) (n=1). (b) Analysis of vimentin expression by western blotting on RPTEC cells using 1 ng/mL of rhIL-15 and 3 ng/mL of rhTGF-β1 for 48h (n=1). Supplementary Figure S3. rhIL-15 treatment did not affect TGF-βR expression, nor Smad2 and Smad3 phosphorylation and nuclear translocation in rhTGFβ1-treated HK-2 cells. (a) Western blot analysis of TGF-βRI and TGF-βRII after 24h or 48h rhIL-15 treatment (1 ng/mL). Bar charts represent TGF-βRI and TGF-βRII expression normalized to GAPDH (n=3, ±SEMs). Antibodies (Abs) against TGFβRI (AF3025) and TGFβRII (AF-241-NA) were obtained from R&D Systems Europe Ltd., Abingdon, UK. (b) Smad2/3 expression and phosphorylation were analyzed by western blotting after a rhTGFβ1 treatment (3 ng/mL, 30 min) in HK-2 cells pretreated or not with rhIL-15 (1 ng/mL, for 24h). Bar charts represent p-Smad2 and p-Smad3 expression normalized to their native form (n=3, ±SEMs). GAPDH is shown as a loading control. Abs against P-Smad2 (400800), Smad2 (511300), P-Smad3 (44246G), and Smad3 (511500) were purchased from Invitrogen (Carlsbad, CA). (c) Smad2 nuclear translocation (SAB4300562, Sigma-Aldrich) was revealed by immunofluorescent staining under the same c [file 9151394.f1.zip › Suppl Figure 2_IJCB_2781291.docx]

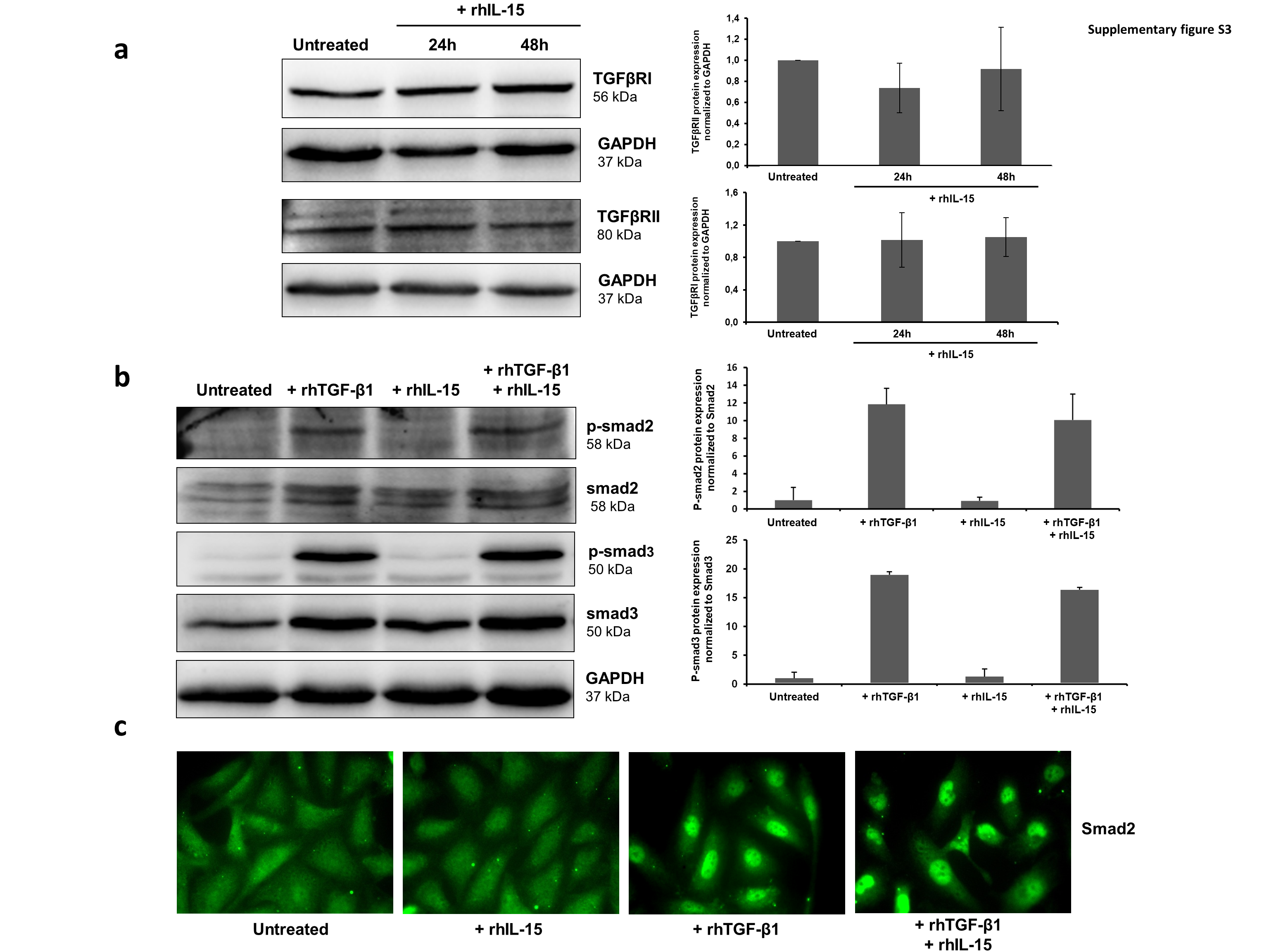

Supplement: Supplementary Materials — Supplementary Figure S1. Expression of IL-15Rβ and IL-15Rγ chains are unaffected along the spontaneous EMT process. Cell surface expression of the IL-15Rβ and IL-15Rγ chains on RPTEC cells was analyzed by flow cytometric analysis after 5 days in the “spontaneous” EMT model. Grey histograms refer to isotype-matched control and black histograms to the expression of IL-15R chains. Mean fluorescence intensity values for each marker are shown in each histogram. Supplementary Figure S2. Inhibition of rhTGF-β1-induced vimentin expression by rhIL-15. (a) Vimentin (mesenchymal marker) expression was analyzed by Western blot at day 5 in RPTEC cells under standard (complete REBM) and “spontaneous EMT” conditions, in presence or absence of neutralizing TGF-β1 antibody (5 μg/mL) and/or rhIL-15 treatment (1 ng/mL) (n=1). (b) Analysis of vimentin expression by western blotting on RPTEC cells using 1 ng/mL of rhIL-15 and 3 ng/mL of rhTGF-β1 for 48h (n=1). Supplementary Figure S3. rhIL-15 treatment did not affect TGF-βR expression, nor Smad2 and Smad3 phosphorylation and nuclear translocation in rhTGFβ1-treated HK-2 cells. (a) Western blot analysis of TGF-βRI and TGF-βRII after 24h or 48h rhIL-15 treatment (1 ng/mL). Bar charts represent TGF-βRI and TGF-βRII expression normalized to GAPDH (n=3, ±SEMs). Antibodies (Abs) against TGFβRI (AF3025) and TGFβRII (AF-241-NA) were obtained from R&D Systems Europe Ltd., Abingdon, UK. (b) Smad2/3 expression and phosphorylation were analyzed by western blotting after a rhTGFβ1 treatment (3 ng/mL, 30 min) in HK-2 cells pretreated or not with rhIL-15 (1 ng/mL, for 24h). Bar charts represent p-Smad2 and p-Smad3 expression normalized to their native form (n=3, ±SEMs). GAPDH is shown as a loading control. Abs against P-Smad2 (400800), Smad2 (511300), P-Smad3 (44246G), and Smad3 (511500) were purchased from Invitrogen (Carlsbad, CA). (c) Smad2 nuclear translocation (SAB4300562, Sigma-Aldrich) was revealed by immunofluorescent staining under the same c [file 9151394.f1.zip › Suppl Figure 3_IJCB_2781292.docx]

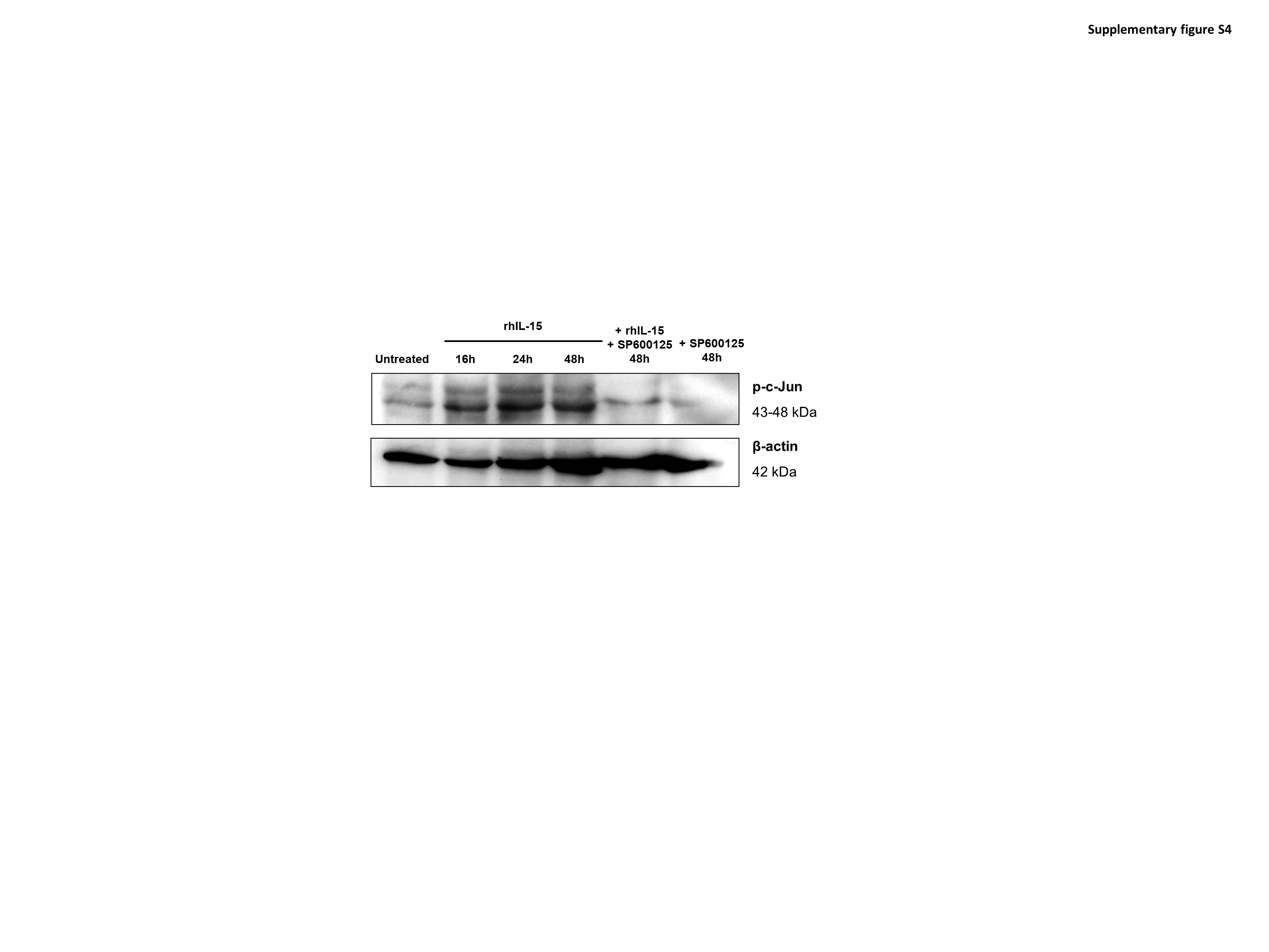

Supplement: Supplementary Materials — Supplementary Figure S1. Expression of IL-15Rβ and IL-15Rγ chains are unaffected along the spontaneous EMT process. Cell surface expression of the IL-15Rβ and IL-15Rγ chains on RPTEC cells was analyzed by flow cytometric analysis after 5 days in the “spontaneous” EMT model. Grey histograms refer to isotype-matched control and black histograms to the expression of IL-15R chains. Mean fluorescence intensity values for each marker are shown in each histogram. Supplementary Figure S2. Inhibition of rhTGF-β1-induced vimentin expression by rhIL-15. (a) Vimentin (mesenchymal marker) expression was analyzed by Western blot at day 5 in RPTEC cells under standard (complete REBM) and “spontaneous EMT” conditions, in presence or absence of neutralizing TGF-β1 antibody (5 μg/mL) and/or rhIL-15 treatment (1 ng/mL) (n=1). (b) Analysis of vimentin expression by western blotting on RPTEC cells using 1 ng/mL of rhIL-15 and 3 ng/mL of rhTGF-β1 for 48h (n=1). Supplementary Figure S3. rhIL-15 treatment did not affect TGF-βR expression, nor Smad2 and Smad3 phosphorylation and nuclear translocation in rhTGFβ1-treated HK-2 cells. (a) Western blot analysis of TGF-βRI and TGF-βRII after 24h or 48h rhIL-15 treatment (1 ng/mL). Bar charts represent TGF-βRI and TGF-βRII expression normalized to GAPDH (n=3, ±SEMs). Antibodies (Abs) against TGFβRI (AF3025) and TGFβRII (AF-241-NA) were obtained from R&D Systems Europe Ltd., Abingdon, UK. (b) Smad2/3 expression and phosphorylation were analyzed by western blotting after a rhTGFβ1 treatment (3 ng/mL, 30 min) in HK-2 cells pretreated or not with rhIL-15 (1 ng/mL, for 24h). Bar charts represent p-Smad2 and p-Smad3 expression normalized to their native form (n=3, ±SEMs). GAPDH is shown as a loading control. Abs against P-Smad2 (400800), Smad2 (511300), P-Smad3 (44246G), and Smad3 (511500) were purchased from Invitrogen (Carlsbad, CA). (c) Smad2 nuclear translocation (SAB4300562, Sigma-Aldrich) was revealed by immunofluorescent staining under the same c [file 9151394.f1.zip › Suppl Figure 4_IJCB_2781293.docx]

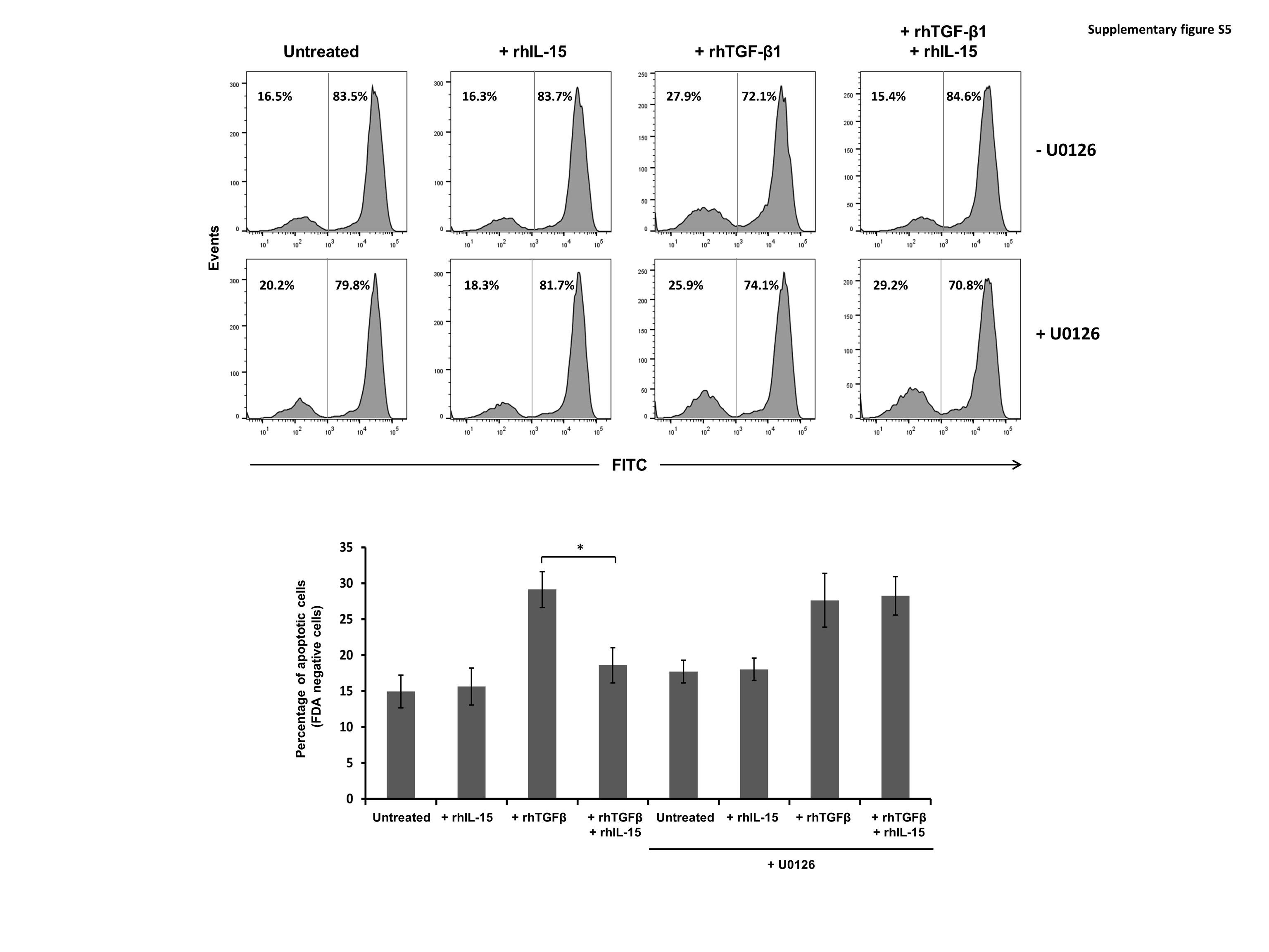

Supplement: Supplementary Materials — Supplementary Figure S1. Expression of IL-15Rβ and IL-15Rγ chains are unaffected along the spontaneous EMT process. Cell surface expression of the IL-15Rβ and IL-15Rγ chains on RPTEC cells was analyzed by flow cytometric analysis after 5 days in the “spontaneous” EMT model. Grey histograms refer to isotype-matched control and black histograms to the expression of IL-15R chains. Mean fluorescence intensity values for each marker are shown in each histogram. Supplementary Figure S2. Inhibition of rhTGF-β1-induced vimentin expression by rhIL-15. (a) Vimentin (mesenchymal marker) expression was analyzed by Western blot at day 5 in RPTEC cells under standard (complete REBM) and “spontaneous EMT” conditions, in presence or absence of neutralizing TGF-β1 antibody (5 μg/mL) and/or rhIL-15 treatment (1 ng/mL) (n=1). (b) Analysis of vimentin expression by western blotting on RPTEC cells using 1 ng/mL of rhIL-15 and 3 ng/mL of rhTGF-β1 for 48h (n=1). Supplementary Figure S3. rhIL-15 treatment did not affect TGF-βR expression, nor Smad2 and Smad3 phosphorylation and nuclear translocation in rhTGFβ1-treated HK-2 cells. (a) Western blot analysis of TGF-βRI and TGF-βRII after 24h or 48h rhIL-15 treatment (1 ng/mL). Bar charts represent TGF-βRI and TGF-βRII expression normalized to GAPDH (n=3, ±SEMs). Antibodies (Abs) against TGFβRI (AF3025) and TGFβRII (AF-241-NA) were obtained from R&D Systems Europe Ltd., Abingdon, UK. (b) Smad2/3 expression and phosphorylation were analyzed by western blotting after a rhTGFβ1 treatment (3 ng/mL, 30 min) in HK-2 cells pretreated or not with rhIL-15 (1 ng/mL, for 24h). Bar charts represent p-Smad2 and p-Smad3 expression normalized to their native form (n=3, ±SEMs). GAPDH is shown as a loading control. Abs against P-Smad2 (400800), Smad2 (511300), P-Smad3 (44246G), and Smad3 (511500) were purchased from Invitrogen (Carlsbad, CA). (c) Smad2 nuclear translocation (SAB4300562, Sigma-Aldrich) was revealed by immunofluorescent staining under the same c [file 9151394.f1.zip › Suppl Figure 5_IJCB_2781294.docx]
